# Prediction of RNA- and DNA-Binding Proteins Using Various Machine Learning Classifiers

**Published:** 2019

**Authors:** Mehdi Poursheikhali Asghari, Parviz Abdolmaleki

**Affiliations:** Department of Biophysics, Faculty of Biological Sciences, Tarbiat Modares University, Tehran, Iran

**Keywords:** DNA-binding proteins, Machine-learning algorithms, RNA-binding proteins

## Abstract

**Background:**

Nucleic acid-binding proteins play major roles in different biological processes, such as transcription, splicing and translation. Therefore, the nucleic acid-binding function prediction of proteins is a step toward full functional annotation of proteins. The aim of our research was the improvement of nucleic-acid binding function prediction.

**Methods:**

In the current study, nine machine-learning algorithms were used to predict RNA- and DNA-binding proteins and also to discriminate between RNA-binding proteins and DNA-binding proteins. The electrostatic features were utilized for prediction of each function in corresponding adapted protein datasets. The leave-one-out cross-validation process was used to measure the performance of employed classifiers.

**Results:**

Radial basis function classifier gave the best results in predicting RNA- and DNA-binding proteins in comparison with other classifiers applied. In discriminating between RNA- and DNA-binding proteins, multilayer perceptron classifier was the best one.

**Conclusion:**

Our findings show that the prediction of nucleic acid-binding function based on these simple electrostatic features can be improved by applied classifiers. Moreover, a reasonable progress to distinguish between RNA- and DNA-binding proteins has been achieved.

## Introduction

Protein-RNA interactions play a fundamental role in different bioprocesses, such as transcription, splicing and translation. RNA-Binding Proteins (RBPs) are pivotal ingredients of RNA splicing, export, stability, localization and translation. They organize all aspects of RNA biogenesis from maturation, surveillance, nucleocytoplasmic transfer to subcellular localization, translation and decomposition 1–3. Therefore, a full comprehension of a diverse variety of cellular processes requires the identification of RBPs.

So far, various computational methods have been developed for the identification of RBPs. Some of them have been based on sequence-derived features such as amino acid composition, dipeptide composition, composition-transition-distribution of seven physicochemical properties, evolutionary information in terms of position-specific scoring matrices and functional domain composition 4–17. The majority of sequence-based methods used the Support Vector Machine (SVM) algorithm for identifying RBPs. On the other hand, alternative methods have utilized the electrostatic features of the protein surface patches in order to identify RBPs 18,19. Moreover, there is a number of structural alignment and fold recognition approaches to tackle with this task 20–24. However, these structure-based methods have a small-scale usage because of limited known structures of proteins. Until now, there are three review papers that have focused specifically and comprehensively on RBPs identification methods 25–27.

Similar to RBPs, DNA-Binding Proteins (DBPs) have been predicted by using two different approaches. In the structure-based approach, the structural alignment and threading-based methods as well as the electrostatic features of the surface patches of proteins have been utilized for identifying DBPs 19,28–36. The electrostatic features have been used in this approach since large positively charged surface patches of proteins usually participate in interaction with DNA molecule. The second approach is the prediction of DBPs based on the sequence information 7,37–52. Numerous machine-learning algorithms have been built based on different encoding schemes of the protein sequence to predict DBPs, in this approach.

Machine-learning algorithms are extensively used to predict the structure and function of proteins 27. Actually, machine learning presents one of the most robust approaches to constructing predictive models in settings where experimentally validated training data are available. At present, however, it is unclear whether the available experimental data regarding DNA-protein and RNA-protein interactions are sufficient for successfully training classifiers using machine learning algorithms 25. Against this fact, this study applies machine learning approaches to train electrostatic-based classifiers for predicting DBPs and RBPs.

This study was done using simple electrostatic features including charge, dipole and quadruple moments for predicting RNA- and DNA-binding proteins by means of neural network method 53. Electrostatic interactions are among the most significant indicators to be considered when one will determine the function of proteins. It is now generally recognized that one must analyze the electrostatic forces in a protein to understand its function 54.

Here, this electrostatic-based approach was extended by applying 9 various machine learning classifiers for identifying RNA- and DNA-binding proteins and also for discriminating RBPs from DBPs. Our goal was to improve the classification accuracy in each of the comparisons. The results demonstrate that this approach can be used by other researchers in this field for more accurate nucleic acid-binding function prediction.

## Materials and Methods

### Datasets

The protein datasets of Ahmad and Sarai 53 were used for our analysis. These datasets consisted of 160 RBP chains, 143 DBP chains and 2441 non nucleic acid-binding protein (Ctrl) chains.

### Electrostatic features

The extracted electrostatic features of Ahmad and Sarai work 53 were used in the current study. These features included the charge, dipole and quadruple moments of protein chains. The detailed description of these features can be found at Ahmad S *et al* 53.

### Machine learning algorithms

In this study, nine classification algorithms of Alternating Decision Tree (ADTree), K-nearest neighbor (K-NN), L1 Regularized Logistic Regression (L1 RLR), L2 Regularized Logistic Regression (L2 RLR), Multilayer Perceptron classifier (MLPClassifier), Random Forest (RF), Radial Basis Function classifier (RBF-Classifier), RealAdaBoost and Sequential Minimal Optimization (SMO) algorithm were used to predict the nucleic acid-binding function of proteins (*i.e*., RNA- and DNA-binding) and also to differentiate between RBPs and DBPs. Waikato Environment for Knowledge Analysis (*WEKA)* package version 3.7.10, an ensemble of machine learning algorithms, was used to perform classifying tasks 55. Below, there is a brief explanation of nine employed classifiers and their corresponding parameter values:

ADTree: It generates an alternating decision tree 55. 20 boosting iterations were used in our experiments. All other parameters of the algorithm were set to default. Our classification was done with *ADTree* function of WEKA.
K-NN: It is a standard non-parametric classification method 55. The basic idea of the K-NN method is that a new case will be classified as the most frequent class among its K-Nearest Neighbors 55. K-NN was used with Euclidean distance and distance weighting (1/distance) and also 75 values of K ranging from K=1 to K=75 were examined and the best ones were selected in terms of the area under receiver operating characteristic curve (AUC) measure. All other parameters of the algorithm were set to default. *IBk* function of WEKA was used for classification.
L1 RLR and L2 RLR: Logistic regression is a well-established method for the classification or prediction of binary response function based on the various independent features. The regression utilizes an objective function and the number of its parameters is as large as the number of features. Usually the objective function contains also a regularization term. It penalizes model details of unnecessary complexity, focuses on the most concerned features, and thus avoids over-fitting of the data used for training (parameter optimization). The most common variants of regularization methods are L1 regularization, also known as Lasso and L2 regularization also known as ridge regression. As penalty term, the L1 regularization adds the sum of the absolute values of the model parameters to the objective function, whereas the L2 regularization adds the sum of squares of parameters 56.

*LibLINEAR* function of WEKA was utilized for doing prediction with these algorithms. The parameter cost (c) was changed from value 1 to 10 and the best one for our classifications was selected. The features were normalized and probability estimates for classification problems were generated. All other parameters of the algorithm were set to default.

MLPClassifier: It trains a multilayer perceptron with one hidden layer 55. *MLPClassifier* function was used and the number of units was changed in the hidden layer and the best architecture for our classifications was selected. All other parameters of the algorithm were set to default.
RF: It is an ensemble classifier method based on decision trees 55. After a large number of trees are generated, each tree in the forest gives a vote for a class and the most popular class among trees for a test instance presents the final classification. A few parameters influence the performance of RF models, such as the number of trees in the forest (ntree) and the number of variables considered at each split (mtry). *Random-Forest* function was used in this study and 500 trees were grown in each experiment. For the number of variables randomly selected at each node, the default value that was equal to the square root of the feature dimension was used. Random forests were trained with a maximum depth of 30 trees.
RBFClassifier: It implements radial basis function networks for classification, trained in a fully supervised manner 55. *RBFClassifier* function was utilized and different numbers of base function were examined and the best one for classification was selected. All other parameters of the algorithm were set to default.
Real AdaBoost: It boosts a two-class classifier using the Real Adaboost method 55. The default parameter values of *RealAdaBoost* function were used for our classifications.
SMO: It is an implementation of SVM algorithm that globally replaces all missing values and transforms nominal attributes into binary ones. It also normalizes all attributes by default. SMO is conceptually simple, easy to implement and faster in computation. Fitting logistic regression models to the outputs of the SMO could in addition provide probability estimates 55.

*SMO* function was used and linear, polynomial (of degree 2) and radial basis function kernels in our classifications were examined and the best ones were selected. The cost factor c was appropriately chosen during the training time. All other parameters of the SMO algorithm were set to default.

### Leave-One-Out Cross-Validation (LOOCV)

The performance of our models trained on Ahmad and Sarai datasets 53 was assessed using LOOCV. In this procedure, one sample was taken out of the whole dataset and was used as the test instance, and the remaining samples were used as training instances. Then, the prediction was made for the test sample. This process was repeated n times (n=total number of samples), and the final performance results were obtained by averaging over all the test results. The sample can be a protein sequence, a protein chain, a DNA sequence, and so on. In this study, the sample was the protein chain. There is not any biological reason for picking this strategy. This is a statistical procedure.

### Performance measures

Various performance measures were used to evaluate the results. These include accuracy, precision, recall, f-measure and the area under Receiver Operating Characteristic (ROC) curve (known as AUC). Accuracy shows proximity of measurement of results to the true value. It can be calculated as [(*TP* +*TN)*/*(TP*+*TN*+*FP*+*FN*)], where *T* refers to true and *F* refers to false, whereas *P* is positive class and *N* is negative class. Recall [(*TP*)/*(TP*+*FN*)] relates to the classifier’s ability to identify positive instances while precision [(*TP)*/*(TP*+*FP*)] is the fraction of predicted instances as positive class that is correctly predicted. F-measure [(2* precision*recall)/(*precision*+*recall*)] is the geometric mean of precision and recall. Also, models based on the ROC curve were evaluated which plot the true positive rate against false positive rate. The AUC value reported by an ROC curve is equal to the probability that a classifier will rank a randomly chosen positive instance higher than a randomly chosen negative one. The AUC is a standard non threshold-dependent index for performance evaluation 57. *ROCR* library 58 of the *R* software 59 version 3.0.1 was used for obtaining numerical values of above-mentioned measures and also drawing ROC curves of different comparisons.

## Results

### RBP chains versus Ctrl chains

Table 1 shows the obtained performance measures of nine employed classification algorithms for differentiation between RBP chains and Ctrl chains, in a LOOCV analysis. The RBFClassifier gave the best classification with the AUC value of 0.850. After that, the K-NN algorithm was the second most robust classifier.

**Table 1.** Performance measures of nine different classification algorithms applied on the RNA-binding protein chains and Ctrl chains, in a LOOCV^k^ analysis

**Classifier**	**AUC^j^**	**F-measure**	**Precision**	**Recall**	**Accuracy**
**ADTree ^a^**	0.828	0.969	0.944	0.996	0.941
**K-NN ^b^**	0.840	0.971	0.951	0.993	0.945
**L1 RLR ^c^**	0.786	0.969	0.939	1.000	0.939
**L2 RLR ^d^**	0.836	0.971	0.949	0.993	0.943
**MLPClassifier ^e^**	0.811	0.968	0.941	0.997	0.939
**RF ^f^**	0.819	0.969	0.941	1.000	0.940
**RBFClassifier ^g^**	**0.850**	0.968	0.939	1.000	0.939
**RealAdaBoost**	0.819	0.968	0.938	1.000	0.938
**SMO ^h^**	0.699	0.969	0.942	0.998	0.940
**NN ^i,l^**	0.780	0.370	0.310	0.450	0.910

a: Alternating Decision Tree;

b: K-Nearest Neighbor;

c: L1 Regularized Logistic Regression;

d: L2 Regularized Logistic Regression;

e: Multilayer Perceptron Classifier;

f: Random Forest;

g: Radial Basis Function Classifier;

h: Sequential Minimal Optimization;

i: Neural Network;

j: Area Under the receiver operating characteristic Curve;

k: Leave-One-Out Cross-Validation;

l: Data obtained from Ahmad and Sarai work 53.

### DBP chains versus Ctrl chains

Table 2 demonstrates the obtained performance measures of nine employed classification algorithms for differentiation between DBP chains and Ctrl chains, in a LOOCV procedure. In the current dataset (*i.e*., DBP and Ctrl chains), the RBFClassifier again presented the best value for AUC (0.852) and then was selected as the best predictor. The MLPClassifer algorithm was the second best classifier with the AUC value of 0.846.

**Table 2.** Performance measures of nine different classification algorithms applied on the DNA-binding protein chains and Ctrl chains, in a LOOCV^k^ procedure

**Classifier**	**AUC ^j^**	**F-measure**	**Precision**	**Recall**	**Accuracy**
**ADTree ^a^**	0.816	0.977	0.957	0.998	0.956
**K-NN ^b^**	0.829	0.972	0.945	1.000	0.945
**L1 RLR ^c^**	0.838	0.972	0.949	0.997	0.947
**L2 RLR ^d^**	0.842	0.972	0.949	0.997	0.946
**MLPClassifier ^e^**	0.846	0.972	0.945	1.000	0.945
**RF ^f^**	0.824	0.972	0.946	0.999	0.946
**RBFClassifier ^g^**	0.852	0.972	0.949	0.997	0.946
**RealAdaBoost**	0.812	0.978	0.957	1.000	0.958
**SMO ^h^**	0.832	0.972	0.945	1.000	0.945
**NN ^i,l^**	0.720	0.220	0.200	0.260	0.900

a: Alternating Decision Tree;

b: K-Nearest Neighbor;

c: L1 Regularized Logistic Regression;

d: L2 Regularized Logistic Regression;

e: Multilayer Perceptron Classifier;

f: Random Forest;

g: Radial Basis Function Classifier;

h: Sequential Minimal Optimization;

i: Neural Network;

j: Area Under the receiver operating characteristic Curve;

k: Leave-One-Out Cross-Validation;

l: Data obtained from Ahmad and Sarai work 53.

### RBP chains versus DBP chains

Finally, table 3 presents the obtained performance measures of nine employed classification algorithms for differentiation between RBP chains and DBP chains, in a LOOCV process. In this dataset, the MLP-Classifer algorithm reached to the value of 0.650 for the AUC that was the highest among other employed algorithms. The RBFClassifer algorithm was the latter in terms of AUC value (0.615).

**Table 3.** Performance measures of nine different classification algorithms applied on the RNA-binding protein chains and DNA-binding protein chains, in a LOOCV^k^ process

**Classifier**	**AUC^j^**	**F-measure**	**Precision**	**Recall**	**Accuracy**
**ADTree ^a^**	0.575	0.715	0.614	0.856	0.640
**K-NN ^b^**	0.609	0.699	0.541	0.988	0.551
**L1 RLR ^c^**	0.605	0.699	0.539	0.994	0.548
**L2 RLR ^d^**	0.607	0.695	0.546	0.956	0.558
**MLPClassifier ^e^**	**0.650**	0.701	0.557	0.944	0.574
**RF ^f^**	0.546	0.697	0.553	0.944	0.568
**RBFClassifier ^g^**	0.615	0.699	0.566	0.913	0.584
**RealAdaBoost**	0.495	0.696	0.533	1.000	0.538
**SMO ^h^**	0.607	0.691	0.528	1.000	0.528
**NN ^i,l^**	0.580	0.690	0.530	1.000	0.530

a: Alternating Decision Tree;

b: K-Nearest Neighbor;

c: L1 Regularized Logistic Regression;

d: L2 Regularized Logistic Regression;

e: Multilayer Perceptron Classifier;

f: Random Forest;

g: Radial Basis Function Classifier;

h: Sequential Minimal Optimization;

i: Neural Network;

j: Area Under the receiver operating characteristic Curve;

k: Leave-One-Out Cross-Validation;

l: Data obtained from Ahmad and Sarai work 53.

### ROC curves

**Figure 1.**
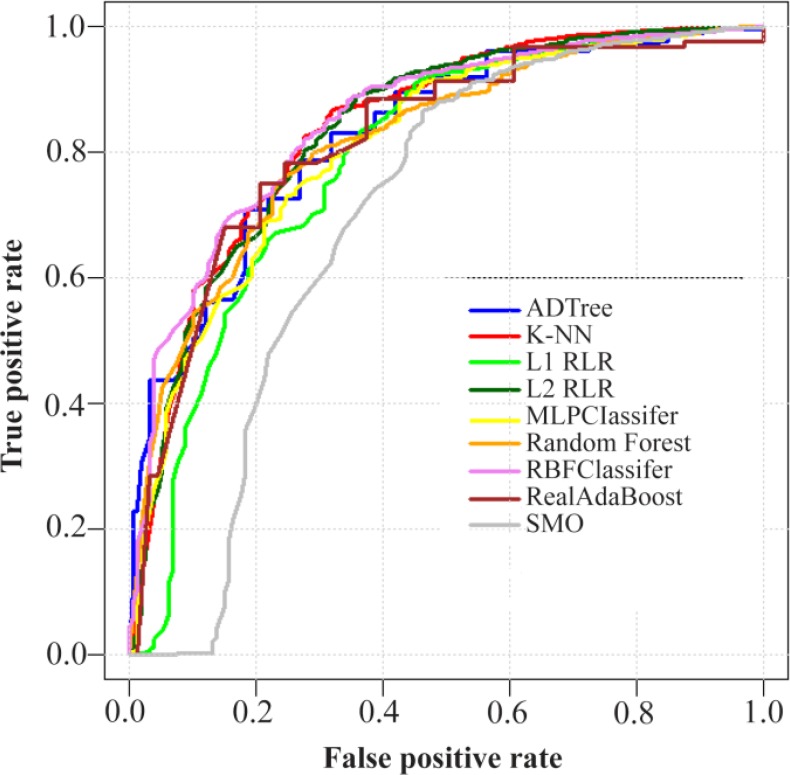
ROC curves of nine machine-learning algorithms employed on RNA-binding protein chains versus ctrl protein chains dataset (consisting of 2601 protein chains) using the LOOCV test. Abbreviations: ADTree, Alternating Decision Tree; K-NN, K-Nearest Neighbor; L1 RLR, L1 Regularized Logistic Regression; L2 RLR, L2 Regularized Logistic Regression; MLPClassifier, Multilayer Perceptron Classifier; RBFClassifier, Radial Basis Function Classifier; SMO, Sequential Minimal Optimization; LOOCV, Leave-One-Out Cross-Validation.

**Figure 2.**
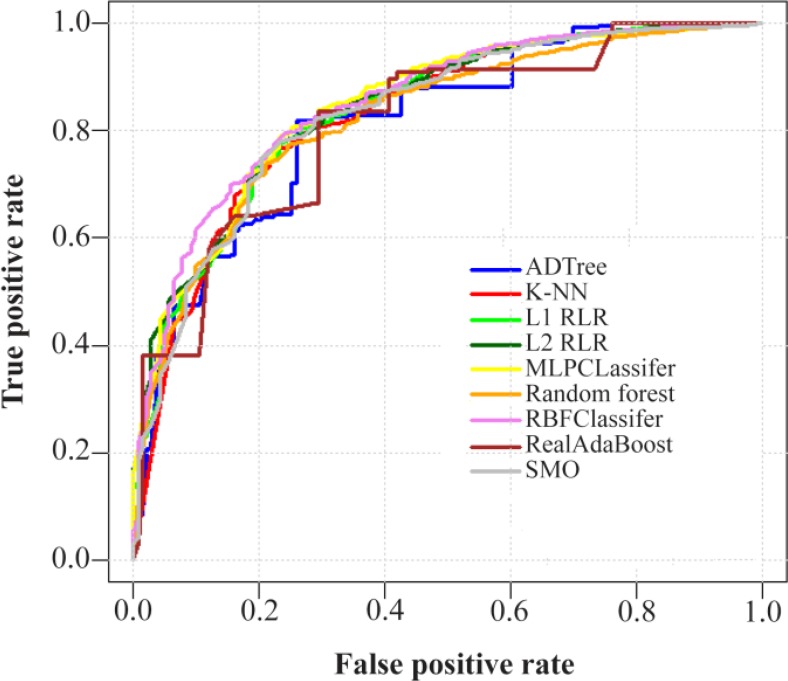
ROC curves of nine machine-learning algorithms employed on DNA-binding protein chains versus ctrl protein chains dataset (consisting of 2584 protein chains) using the LOOCV test. Abbreviations: ADTree, Alternating Decision Tree; K-NN, K-Nearest Neighbor; L1 RLR, L1 Regularized Logistic Regression; L2 RLR, L2 Regularized Logistic Regression; MLPClassifier, Multilayer Perceptron Classifier; RBFClassifier, Radial Basis Function Classifier; SMO, Sequential Minimal Optimization; LOOCV, Leave-One-Out Cross-Validation.

**Figure 3.**
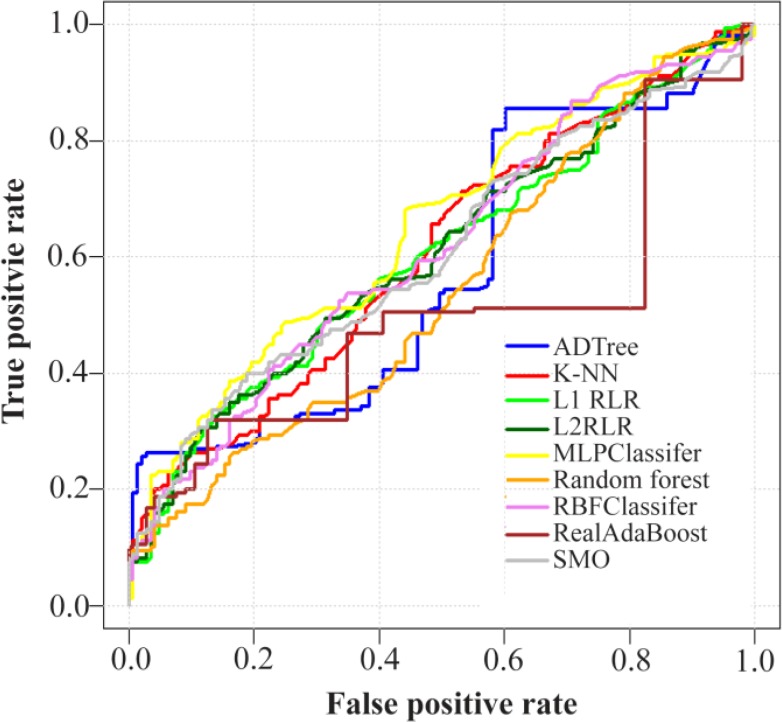
ROC curves of nine machine-learning algorithms employed on RNA-binding protein chains versus DNA-binding protein chains dataset (consisting of 303 protein chains) using the LOOCV test. Abbreviations: ADTree, Alternating Decision Tree; K-NN, K-Nearest Neighbor; L1 RLR, L1 Regularized Logistic Regression; L2 RLR, L2 Regularized Logistic Regression; MLPClassifier, Multilayer Perceptron Classifier; RBFClassifier, Radial Basis Function Classifier; SMO, Sequential Minimal Optimization; LOOCV, Leave-One-Out Cross-Validation.

For better comparison of applied machine-learning algorithms on 3 datasets, their corresponding ROC curves were depicted (Figures 1-3).

### Comparison with other methods

The results of this research are comparable with the results of Ahmad and Sarai 53, because their protein datasets and also their extracted features were used in this study. The difference between their work and the current study is utilizing nine machine-learning algorithms other than the neural network they employed.

Obtained results in this study show improvement in the performance measures, especially in the AUC measure, when comparing with their work. Ahmad and Sarai reached to the AUC value of 0.78 for RBPs versus ctrl dataset, while this measure improved by 0.07 by means of RBFClassifier algorithm in the current study. Likewise, the accuracy was improved by 0.03 and reached to the 0.94. Another algorithm which improved these measures was K-NN which raised the AUC and accuracy values up to 0.84 and 0.94, respectively. Among 9 machine-learning algorithms employed on this dataset, only SMO algorithm had the AUC value less than neural network method. However, this algorithm had the accuracy value (0.94) more than that of Ahmad and Sarai method.

In the second dataset, *i.e*., DBPs *vs*. ctrl, all of the nine utilized machine-learning algorithms yielded the AUC values more than the ones in Ahmad and Sarai work. The AUC value was interestingly improved by 0.13 and reached to the value of 0.85 by means of RBFClassifier, in comparison with the AUC value of their method (0.72). Also, the accuracy value was improved by all algorithms.

In the third comparison, *i.e*., RBPs *vs*. DBPs, the AUC value was raised to 0.65 by MLPClassifer and showed an increase by 0.07 compared to their obtained value (0.58). The precision measure was improved by the ADTree algorithm and reached to the value of 0.61, but the AUC value of this algorithm (0.575) was less than that of neural network method (0.58).

## Discussion

In this study, 9 machine-learning algorithms were used for discrimination between RBPs and ctrl chains, DBPs and ctrl chains and finally between RBPs and DBPs. The obtained results demonstrated that our selected classification algorithms can further improve predictions constructed with only 5 electrostatic features obtained from low-resolution protein structures. Then, these features show the capability for nucleic acid-binding function prediction and also for discriminating RNA-binding from DNA-binding function. Adding more informative features to the prediction process can improve the performance measures and hence increase the classification accuracy.

Two robust classifiers in 3 different comparisons were RBFClassifer and MLPClssifier. These two classifiers have architectures similar to neural network. Therefore, similar to Ahmad and Sarai work 53, neural network methods show significant robustness for prediction of nucleic acid-binding function with the help of electrostatic features.

The importance of this work is to support this idea that simple electrostatic features such as charge, dipole and quadruple moments are useful for identification of nucleic acid-binding function. These electrostatic features can be combined with other sequence or structure-based features for precise function prediction at the level of nucleic acid-binding. Also, the discrimination between RNA- and DNA-binding function is reinforced by these features as well as other suitable ones.

Given the relatively small sizes of the nucleic acid-binding proteins analyzed in this study, discrepancies in the results obtained using different classifiers to predict nucleic acid-binding proteins must be interpreted with caution. It will be important to evaluate these methods on larger, more complete datasets of experimentally validated nucleic acid-binding proteins as they become available.

### Notes for practical implementation

**Figure 4.**
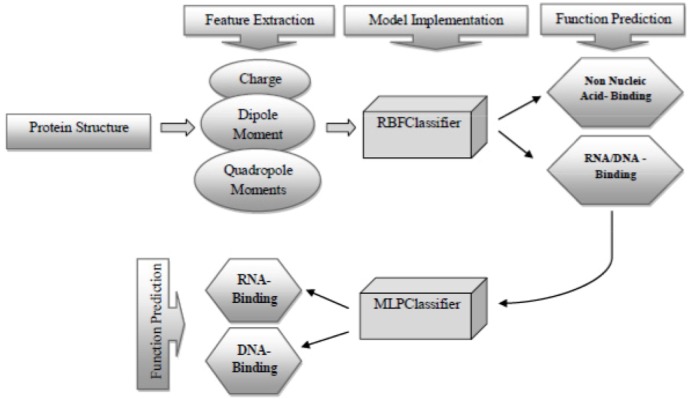
The overall workflow for practical implementation. Firstly, the query protein is represented numerically by three kinds of features. Secondly, the first round of the classification is done using the best-selected classifier trained on combined full dataset (i.e. the RBFClassifier). Thirdly, if the function of query protein was predicted as nucleic acid-binding (or RNA/DNA-binding), the second round of the classification is attempted based on the best-selected classifier trained on the nucleic acid-binding proteins dataset (*i.e*. the MLPClassifier). The final predicted function identifies the query protein as either RNA-binding or DNA-binding. Abbreviations: MLPClassifier, Multilayer Perceptron Classifier; RBFClassifier, Radial Basis Function Classifier.

The overall workflow for practical implementation of our method is shown in figure 4. Given the protein structure as the input, five electrostatic features are extracted from the query protein. Then, the RBFClassifier as the most powerful method in discriminating between RNA/DNA-binding and non-nucleic acid-binding proteins uses its trained structure to determine whether the query is a RNA/DNA-binding protein or not. If the protein function was predicted as nucleic acid-binding (or RNA/DNA-binding), then the classification continues to distinguish RNA-binding function from DNA-binding one utilizing the trained structure of MLP-Classifier, *i.e*. the best-selected classifier trained on the nucleic acid-binding proteins dataset.

## Conclusion

Nine different machine-learning algorithms other than neural network with a higher capability for nucleic acid-binding function prediction have been introduced. These classifiers showed reasonable improvement that highlights their potential to be used by other researchers for nucleic acid–binding function prediction. It is hoped that the use of alternative sequence or structure-based features as well as the electrostatic features will reinforce nucleic acid-binding function prediction protocols.
